# Multifunctional Biocatalysts for Organic Synthesis

**DOI:** 10.1021/jacs.3c09542

**Published:** 2024-03-15

**Authors:** Thomas W. Thorpe, James R. Marshall, Nicholas J. Turner

**Affiliations:** † Department of Chemistry, University of Manchester, Manchester Institute of Biotechnology, 131 Princess Street, Manchester, United Kingdom, M1 7DN

## Abstract

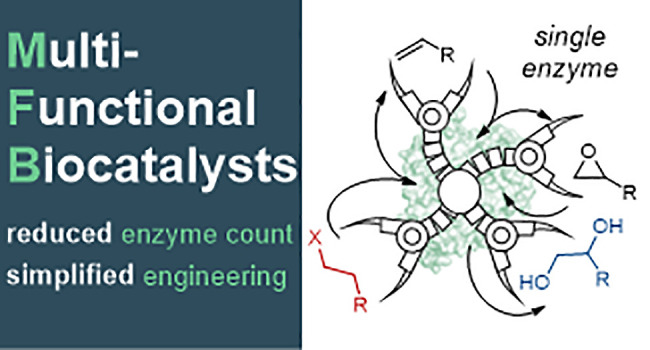

Biocatalysis is becoming an indispensable tool in organic synthesis due to high enzymatic catalytic efficiency as well as exquisite chemo- and stereoselectivity. Some biocatalysts display great promiscuity including a broad substrate scope as well as the ability to catalyze more than one type of transformation. These promiscuous activities have been applied individually to efficiently access numerous valuable target molecules. However, systems in which enzymes possessing multiple different catalytic activities are applied in the synthesis are less well developed. Such multifunctional biocatalysts (MFBs) would simplify chemical synthesis by reducing the number of operational steps and enzyme count, as well as simplifying the sequence space that needs to be engineered to develop an efficient biocatalyst. In this Perspective, we highlight recently reported MFBs focusing on their synthetic utility and mechanism. We also offer insight into their origin as well as comment on potential strategies for their discovery and engineering.

Enzymes display remarkable catalytic activity, evolvability and reaction promiscuity, features that have resulted in their widespread application as biocatalysts in chemical synthesis.^1^ Promiscuity is manifested through broad substrate scope, as well as the capacity for a single enzyme to catalyze mechanistically different reactions. This reaction promiscuity is clearly relevant to the evolution of enzymes and provides a stimulus for the design of new enzyme activities going forward. Another feature of some enzymes is the ability to catalyze two or more chemically distinct reactions during the overall conversion of substrate to product. Such multifunctional biocatalysts (MFBs) would complement similar recent developments in other branches of catalysis including homogeneous and heterogeneous catalysis.^2,3^ MFBs could have a major impact on cascade biocatalysis, reactions that currently require the combination of several biocatalysts to perform multiple chemical transformations in one-pot. To apply cascades in chemical manufacturing, each biocatalyst is typically engineered separately, followed by process optimization to ensure enzyme compatibility and high productivity.^4,5^ MFBs could make cascade processes more efficient by reducing the enzyme count and hence catalyst loading as well as the number of operational steps (Scheme 1). Furthermore, these biocatalysts could simplify enzyme engineering by reducing the sequence space needed to be explored. However, improving MFBs by coevolving multiple encoded activities will likely require new strategies guided by detailed mechanistic understanding.

**Scheme 1:**
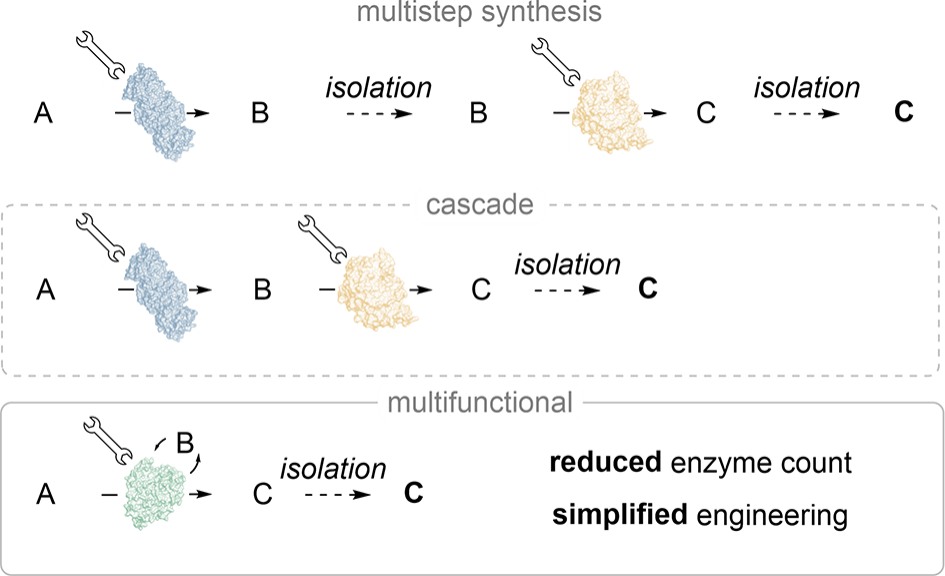
Multifunctional Biocatalysts (MFBs) Simplify Chemical Synthesis and Biocatalyst Development for the Conversion of A to B to C

The ability of an enzyme active site to catalyze two or more mechanistically distinct reactions presents a number of significant challenges, in particular: (i) the ability to bind and orientate structurally related molecules since the product from each reaction step becomes the substrate for the next; (ii) the availability of catalytic active-site residues (*e.g*., proton donors, proton acceptors, nucleophiles) that can participate in different nonbonding interactions; and (iii) in some cases the availability of (multiple) cofactors (*e.g*., NAD(P)H, FADH_2_) that can mediate different processes *e.g*. reduction of C=O as well as C=N bonds.

This Perspective highlights recent MFBs that specifically catalyze two or more chemically different reactions during the conversion of substrate to product within a single active site. MFBs not described in this perspective, that are also valuable but may not possess all the outlined advantages, are enzymes that possess multiple active sites,^6^ including natural and engineered tethered/fusion multidomain biocatalysts.^7^ The examples below include discussion of the underpinning mechanistic basis for their multifunctionality together with their synthetic utility. Additionally, we comment on the origins of these biocatalysts and consider potential strategies for discovering and engineering more complex MFBs.

## 4-OT Can Exploit Different Reactive Amine-Intermediates

**Scheme 2:**
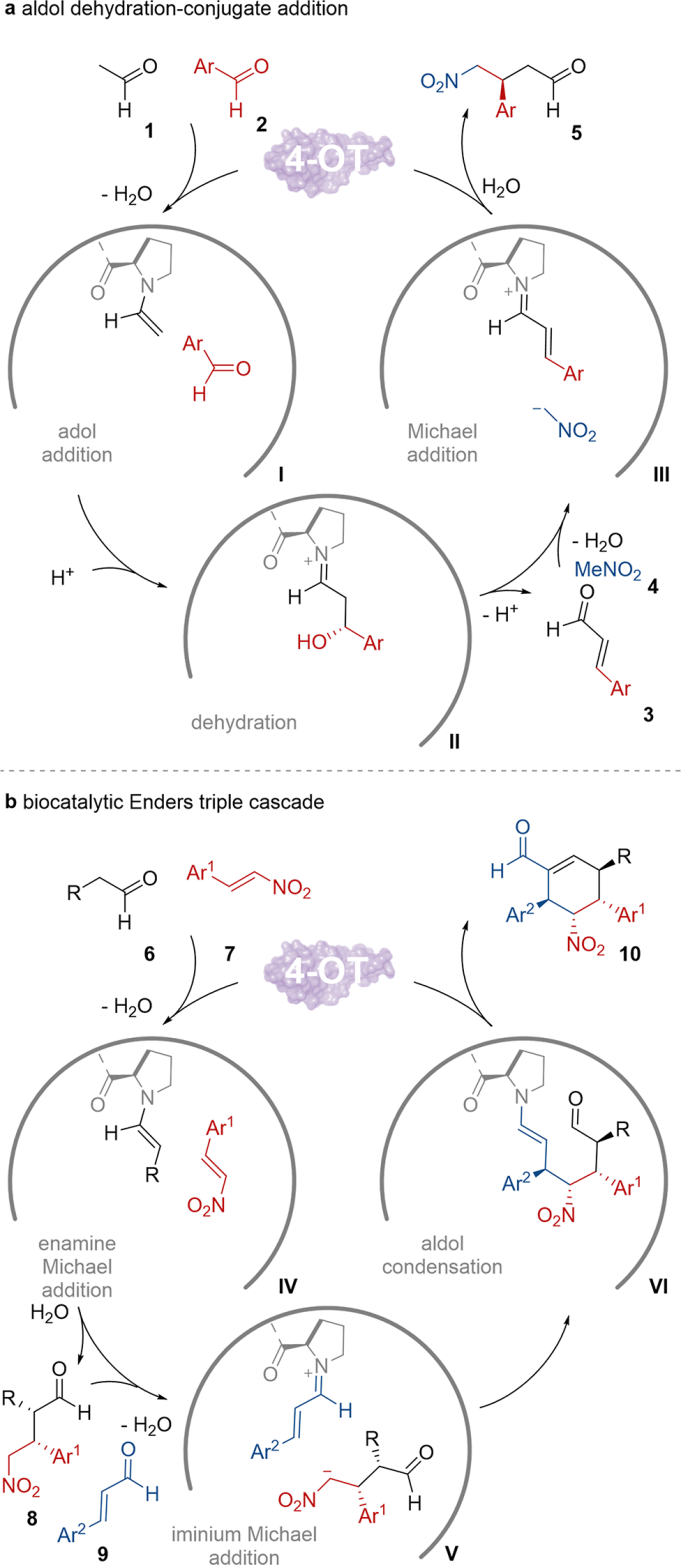
Catalytic Cycles of 4-OT Multifunctional Reactions: (a) Aldol Dehydration-Conjugate Addition; (b) Three-Component Cyclization Cascade

At 62 amino acids, 4-oxalocrotonate tautomerase (4-OT) is one of the smallest known enzyme subunits. Despite its size, 4-OT is a highly active and promiscuous enzyme with respect to substrate scope and the types of catalyzed reactions.^8^ The Poelarends group have extensively studied 4-OT demonstrating epoxidation,^9^ cyclopropanation,^10^ aldol condensation^11^ and hydration reactions.^12^ Several of these activities were combined to create a one-pot cascade for the conversion of aldehydes **1** and **2** to nitroaldehydes **5** using 4-OT as a single MFB (Scheme 2a).^13^ As with many examples of MFBs, the authors were inspired by chemocatalysis, in this case aminocatalysts.^14^ In this sequence, 4-OT initially forms a nucleophilic enamine intermediate **I** between aliphatic aldehyde **1** and the N-terminal proline to allow aldol addition to aromatic aldehyde **2**, before catalyzing the dehydration of intermediate **II** to the corresponding α,β-unsaturated aldehyde **3**. This alkene is further activated by the same proline residue as an electrophilic unsaturated iminium intermediate **III** for subsequent nitromethane **4** Michael addition to reveal γ-nitroaldehyde **5**. By employing the 4-OT variant F50A, identified by spectrophotometrically screening almost all possible single mutant variants of 4-OT, a one-pot reaction combining these three reactivities could be developed. This system was used to generate γ-nitroaldehydes **5** with a few functionalized aromatic substitutions that were oxidized by an aldehyde dehydrogenase to generate the corresponding γ-nitrocarboxylic acids in up to 80% yield with 99% ee.

More recently, enamine and iminium carbonyl activation by 4-OT has been further explored in the development of biocatalytic reactions that created up to 3 new C–C bonds using a single enzyme.^15^ The authors designed a tandem-fused 4-OT variant from *Pseudomonas putida* containing an internal His-Tag, Pp-4-OT-F_3_, for remarkable two- and three-component reactions for the synthesis of densely functionalized cyclohexene carbaldehydes **10**. In the two-component cascade system, various electron-rich and -poor cinnamaldehydes combined with nitromethane **4** could be converted by an iminium–iminium-enamine sequence to cyclohexene carbaldehydes **10**. While the products were obtained with good to high yields, exquisite enantioselectivity, and mostly excellent diastereoselectivity, this system was limited to species in which Ar^1^ = Ar^2^ without using a second enzyme (see Scheme 2b for product). The three-component reaction sequence (Scheme 2b), which mirrors Enders’ organocatalytic cascade,^16^ involves a sequence of enzymatic Michael addition of aldehyde **6** to a nitroalkene **7** via enamine intermediate **IV**. Next, a second Michael addition of the product of the first step **8** with an iminium-activated intermediate **V** of unsaturated aldehyde **9** reveals the enamine complex **VI** that undergoes biocatalytic intramolecular aldol addition and dehydration to form the final carbocycle **10**. Initial reactions using the Pp-4-OT fusion resulted in the isolation of **8** only. Evaluation of the stereochemistry of this intermediate revealed a matched-mismatched effect as the enzyme accommodates only the (*S*)-enantiomer of **8** in the iminium-Michael step **V** which is formed with (*R*)-selectivity in the enamine Michael step **IV**. The author’s strategy to overcome this mismatch utilized enzyme engineering to create the variant Pp-4OT-F_3_^M117Y/F122A^ that was able to produce the required stereoisomer in the first step while maintaining activity for the latter steps. This variant was able to form several diversely substituted cyclohexene carbaldehydes **10** with up to 4 stereocenters, 51% yield, >99% ee and >20:1 dr.

## Flavin-Dependent ‘Ene’-Reductases Are MFBs That Possess Diverse Multifunctionality

**Scheme 3:**
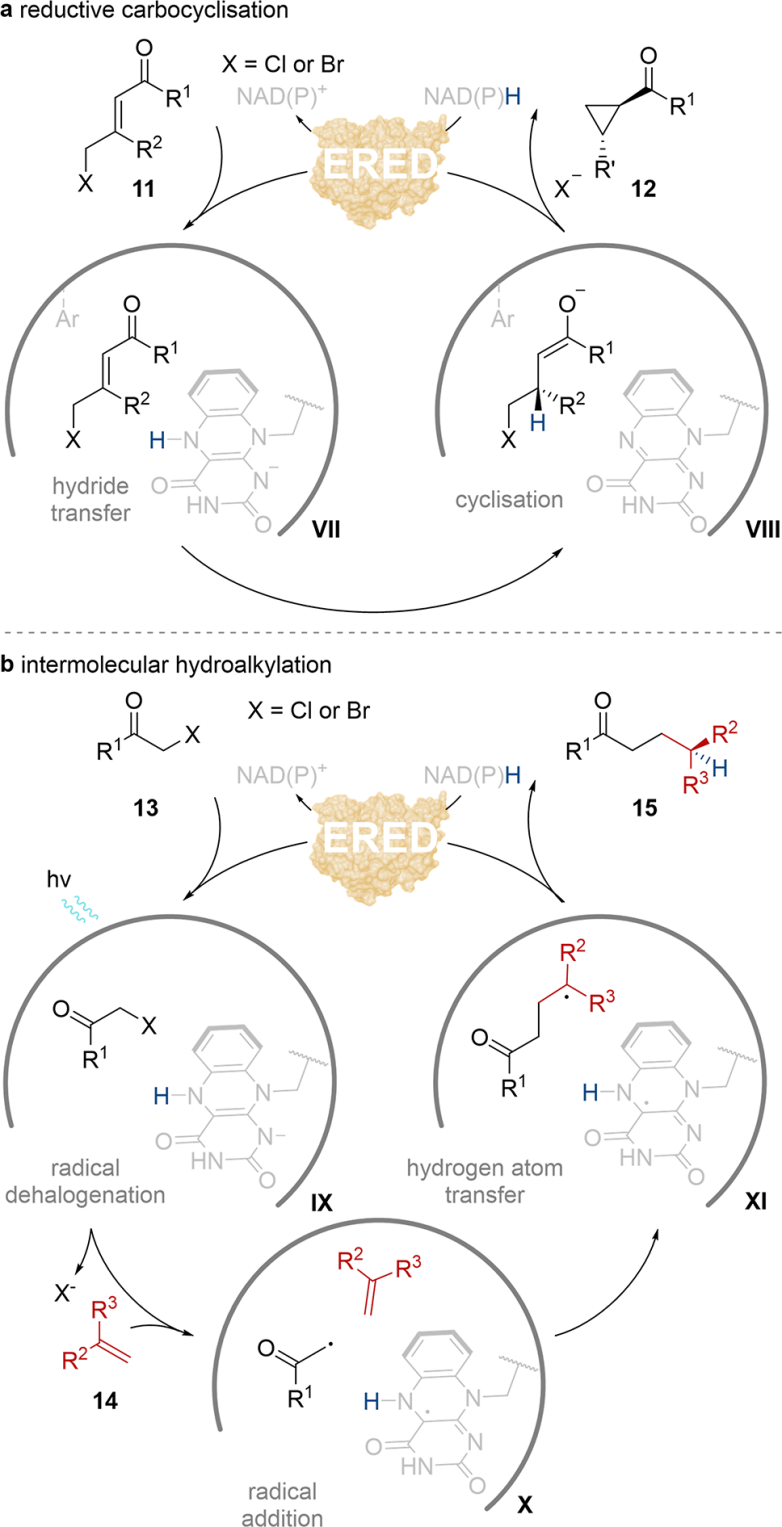
Catalytic Cycles of ERED Multifunctional Reactions: (a) Asymmetric Reductive Carbocyclization; (b) Asymmetric Intermolecular Photobiocatalytic Hydroalkylation *via* Tertiary Electron Donor–Acceptor Complex

Flavin mononucleotide (FMN)-dependent ‘ene’-reductases (EREDs) are highly promiscuous and catalyze various mechanistically distinct reactions including their well characterized hydrogenation of electronically activated alkenes.^17−19^ The mechanism for this conjugate reduction is itself a multifunctional process as both hydride transfer from FMN_hq_ and stereoselective protonation are ERED-catalyzed.^20^ Heckenbichler et al. exploited this MFB mechanism by intercepting the enolate-type intermediate with electrophiles to develop a C–C bond forming reaction (Scheme 3a).^21^ Suitable alkene substrates containing a pendant ω-halogen **11** allowed a new carbocyclization reaction pathway following hydride delivery to complex **VII** to compete with the canonical protonation step. To enhance the efficiency of this alkylation pathway, variants possessing hydrophobic amino acid side chains (Phe and Trp) in place of the protonation residue (Tyr) were created and allowed the reductive carbocyclization of intermediate **VIII** to predominate and form the corresponding stereoenriched aldo- and keto-cyclopropanes **12** with up to 5:95 reduction:cyclization, >99% conversion, >99% ee and 97:3 dr.

More recently, EREDs have been explored as MFBs for controlling single electron processes initiated by enzyme catalyzed irradiative dehalogenation. ERED radical reactions that have been developed include hydrodehalogenation of α-bromoesters,^22^ redox-neutral radical cyclization of α-halo-β-amidoesters,^23^ and sp^3^–sp^3^ cross-electrophile coupling.^24^ However, a key development in this area has been asymmetric reductive C(sp^3^)–C(sp^3^) hydroalkylation. Initial studies established intramolecular hydroalkylations as an ERED controlled process in which each reaction step occurs within a single active site.^25,26^ Further developments demonstrated the first intermolecular variant of this reaction, the hydroalkylation of α-halocarbonyls **13** and alkenes **14**.^27^ This system follows a similar mechanism (Scheme 3b). Light irradiation of ERED-substrate-FMN_hq_ electron donor–acceptor complex **IX** promotes single electron transfer from the flavin, before mesolytic cleavage of the C–halogen bond of **13** to generate an electrophilic radical intermediate **X**. The ERED then controls the trajectory of this radical such that addition to alkene 1**4** occurs before hydrogen atom transfer to the resulting prochiral radical intermediate **XI** from FMN_sq_. In this system, Y196, the conventional proton source in ERED conjugate alkene reduction, mediates the terminating step and results in experimentally observed (*S*)-γ-enriched carbonyl **15**. This system was applied in the coupling of α-bromoesters, amides and ketones with various α-alkyl aryl substituted ethylenes with up to 99% yield and 99% ee. Aliphatic alkenes as well as α-chlorocarbonyls and an α-methyl enyne were all accepted as substrate partners but resulted in lower enantioselectivities or yields respectively. Additionally, secondary α-bromo carbonyls could each be coupled with α-methyl styrene with high enantioselectivity but exhibited poor diastereocontrol.

A concurrent study that examined different EREDs revealed a different substrate scope as well as additional mechanistic insights into the control of reaction route.^28^ A broad selection of α-chloro carbonyls **13**, predominately tertiary amides, were effectively coupled with terminally disubstituted alkenes **14** in up to 99% yield and 98% ee. Aryl alkenes were well accepted as substrates in addition to aliphatic and heteroatom substituted alkenes, but the latter suffered from lower enantioselectivities. UV–vis spectroscopy and labeling experiments, designed to identify respectively potential electron donor–acceptor complexes and reveal the termination step, highlighted differences in reaction mechanism compared to the prior hydroalkylation study. The investigated MFBs formed a quaternary rather than tertiary electron donor–acceptor complex consisting of ERED, α-chlorocarbonyl, alkene, and FMN_hq_ to facilitate C–halogen bond cleavage and favor coupling over hydrodehalogenation. Furthermore, labeling experiments revealed that termination appeared to occur nearly exclusively as a direct transfer from FMN_sq_.

## P450s Repurposed as Multifunctional Catalysts

**Scheme 4:**
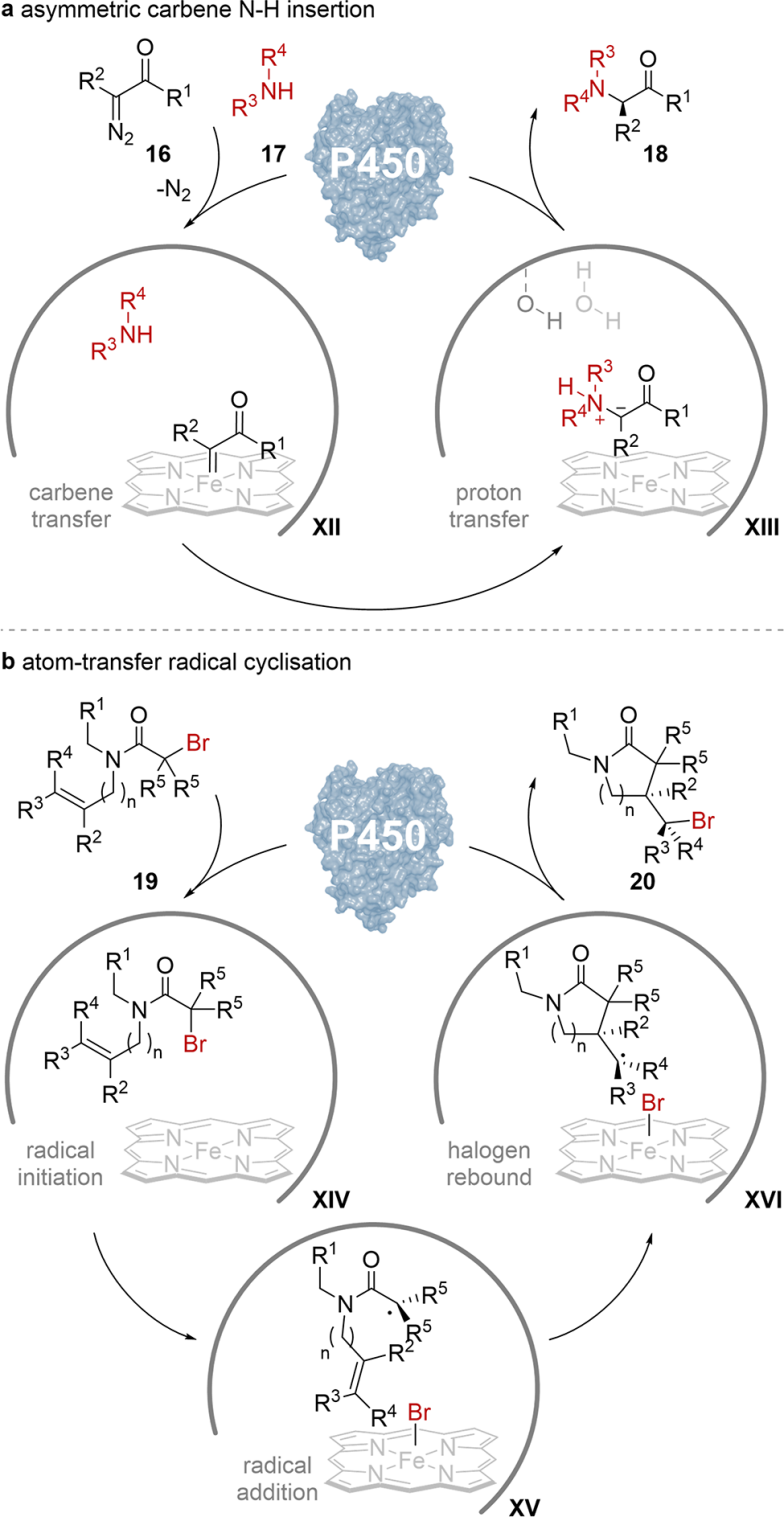
Catalytic Cycles of Multifunctional P450 Reactions: (a) Asymmetric Carbene N–H Insertion; (b) Atom-Transfer Radical Cyclization

Cytochrome P450 monooxygenases (P450s) are enzymes that catalyze a diverse range of chemical transformations at their iron-heme containing active site, ranging from oxygenation to oxidative aryl–aryl cross-coupling.^29,30^ Directed evolution of P450s has enabled the formation of new reactive enzyme-intermediates for diverse carbene and nitrene transfer reactions.^30^ The asymmetric insertion of carbenes into N–H bonds is a particularly challenging multistep process, as a MFB must catalyze the nucleophilic addition of an amine to a metal-carbene intermediate as well as the protonation of the resulting ylide (Scheme 4a).^31^ Inspired by chemocatalytic strategies for this reaction that utilize complementary carbene transfer and proton transfer catalysts, Liu et al. were able to identify previously engineered P450_BM3_ variants that were not only highly active but were also able to precisely control the protonation step by complex **VII**.^32^

Characterization of the identified enzyme lineage and point variation studies revealed that the A264S mutation significantly improved the enantioselectivity of the insertion reaction, as well as enzymatic activity. Molecular dynamics simulations suggested that S264 contributes to the improved reactivity by increasing the electrophilicity of the lactone-carbene in intermediate **VI**, as well as restricting the active site substrate orientation from which amine **17** addition produces the ylide. Subsequent, rapid protonation of this intermediate from a precisely placed active site water molecule reveals the amine product **18**. The heme-protein was able to couple a broad selection of aromatic, aliphatic, primary, and secondary amines in 1 to 1 equivalence with a diazo-lactone **16** in up to >99% yield and 98% ee.

P450s have also been repurposed as MFBs for asymmetric atom-transfer radical cyclization (ATRC).^33^ This redox neutral reaction is widespread in conventional synthesis for building molecular complexity, yet, prior to this study, stereoselective ATRC was rare, as small molecule catalyst-radical intermediate association is difficult to maintain during the stereoselective steps.^34^ To realize an asymmetric ATRC, a variety of Fe-dependent proteins were screened, and stereocomplementary P450 variants were identified. These MFBs catalyze the sequential, ground state single electron transfer from iron-heme to organic halide **19**, then radical cyclization across a pendant alkene through intermediate **XV**, and final bromine rebound via complex **XVI** to regenerate the biocatalyst. While the initial P450s presented low enantioselectivity for the conversion, iterative site-saturation mutagenesis yielded mutants with superior stereoselectivity and total turnover number and facilitated the synthesis of several stereoenriched 4- to 6-membered *N*-substituted lactams **20** with up to 81% yield, 98% ee, and 24:1 dr. Subsequent computational and mutagenesis studies further examining the mechanism of P450 ATRC has revealed that the orientation of the radical cyclization transition state and not ground state substrate is responsible for enantioselectivity of this step.^35^ Furthermore, an important mutation, I263Q, could be identified that facilitates substrate binding, acceleration of the rate-determining radical initiation step, and the anchoring of intermediate **XV** for the stereocontrolled cyclization.

## Ene-IREDs Sequentially Reduce Different Functional Groups Using the Same Cofactor

**Scheme 5:**
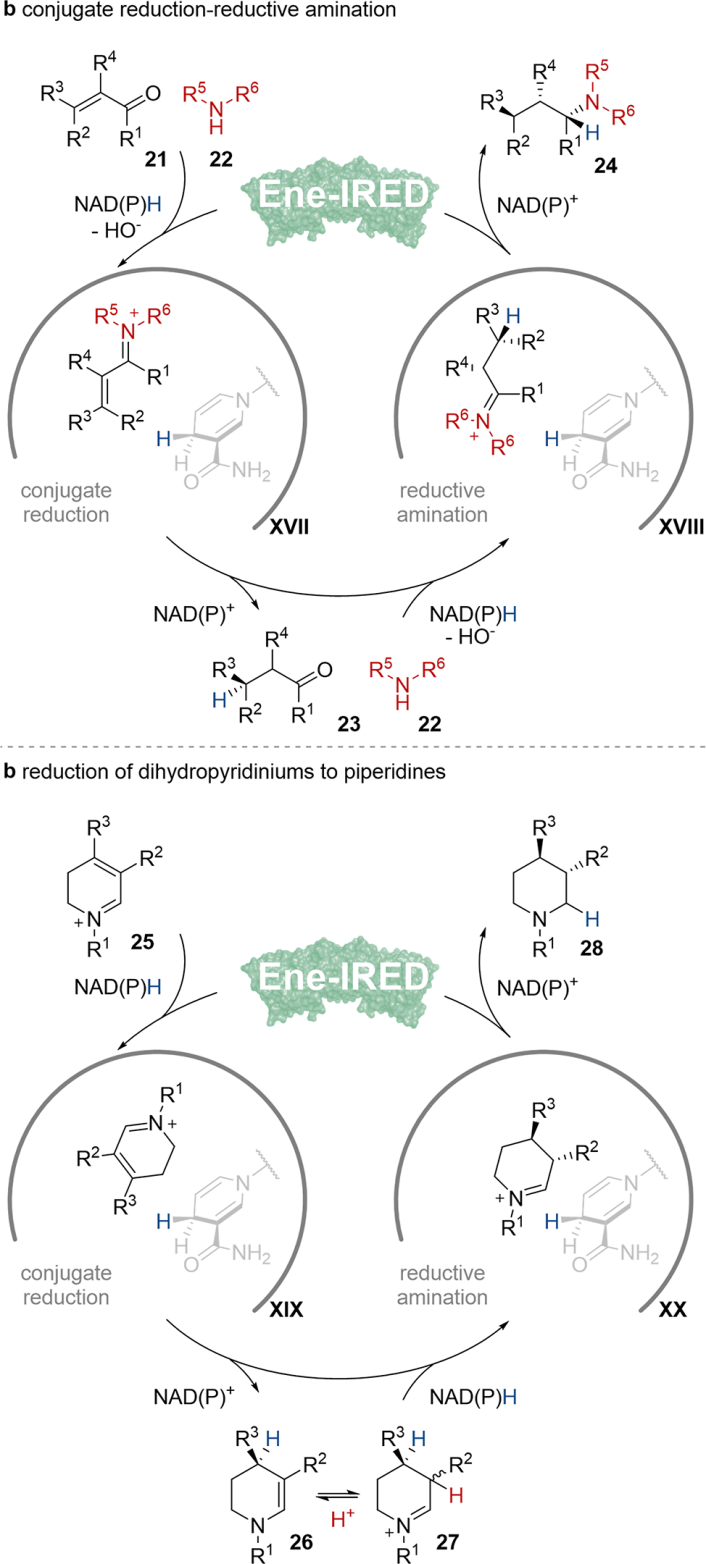
Catalytic Cycles of Ene-IREDs Multifunctional Reactions: (a) Conjugate Reduction-Reductive Amination; (b) Reduction of Dihydropyridiniums to Piperidines

Imine reductases (IREDs) have emerged as broadly applicable biocatalysts for the synthesis of a wide range of chiral amines including high value pharmaceuticals and agrochemicals.^36^ IREDs have broad substrate scope and are catalytically promiscuous for imine reduction,^37^ reductive amination,^38^ and in certain cases activated ketone reduction.^39^ We reported the first examples of IREDs, that catalyze conjugate alkene reduction and then reductive amination or imine reduction, and termed these enzymes as ene-imine reductases (Ene-IREDs).^40^ The first Ene-IRED was identified by chromatographically screening a metagenomic IRED collection^41^ for the complete reduction of a cyclic ene-imines. The selected enzyme was then applied in conjugate reduction-reductive amination of unsaturated carbonyls **21** with amines **22** (Scheme 5a). Ene-IRED was able to couple a broad selection of 2- or 3-substituted enals and enones with sterically unhindered primary and cyclic secondary amines to selectively prepare numerous chiral secondary and tertiary amines **24** containing up to three stereogenic centers with up to 80% yield, >99% ee and 99:1 dr. Mechanistic, structural and mutagenesis studies established a reaction sequence of conjugate reduction followed by reductive amination deriving the hydrides from NADPH and identified important residues within the active site that influence reactivity and stereocontrol in both steps. Removing the amine substrate partner **22** from the reaction led to the complete abolition of any reactivity, indicating that iminium activation of alkene **21** is required for the conjugate reduction step. These studies established two separate EneIRED catalytic cycles, conjugate reduction and reductive amination, that work synergistically to generate the amine product via two different iminium complexes, **XVII** and **XVII** respectively.

More recently, we utilized EneIRED conjugate reduction-imine reduction activity within an efficient system for the asymmetric dearomatization of activated pyridines to piperidines **28** (Scheme 5b).^42^ Here, following an initial chemical reduction of *N*-alkylpyridiniums to tetrahydropyridines, dihydropyridiniums **25** are generated *in situ* by an amine oxidase for subsequent full reduction by EneIREDs to stereoenriched piperidines **28** via ene-iminium and iminium complexes respectively, **XIX** and **XX**. By rescreening the metagenomic IRED panel^41^ for EneIRED activity in combination with a 6-hydroxy-d-nicotine oxidase variant, two series of enzymes, including the original EneIRED, could be identified that gave access to both C-3 enantiomeric sets of piperidine **28**. Using this chemo-enzymatic dearomatization a diverse array of 3-, 4- and 3,4-substituted piperidines could be prepared with up to 89% yield, >99% ee and 90:10 dr. The system tolerated heterocyclic and sterically demanding groups, as well as species with synthetic handles for downstream chemistry. Furthermore, the antipsychotic drug, OSU-6162, could be prepared directly using the system, as well as synthetic intermediates to therapeutics Preclamol and Niraparib. The reaction mechanism was investigated using *in situ* reaction monitoring, deuteration labeling experiments, and structural studies. Notably the isolation of a tetrahydropyridinium **27** (R^1^ = Et, R^2^ = naphthyl R^3^ = H) from an amine oxidase-EneIRED reaction, as well as studies of the reaction of this species with EneIRED only, suggested that **26** is an intermediate in the reaction pathway and that C-3 stereochemistry of the piperidine product **28** is set by an EneIRED controlled dynamic kinetic resolution through rapid epimerization via **27**.

## Discovery and Creation of New MFBs

**Scheme 6:**
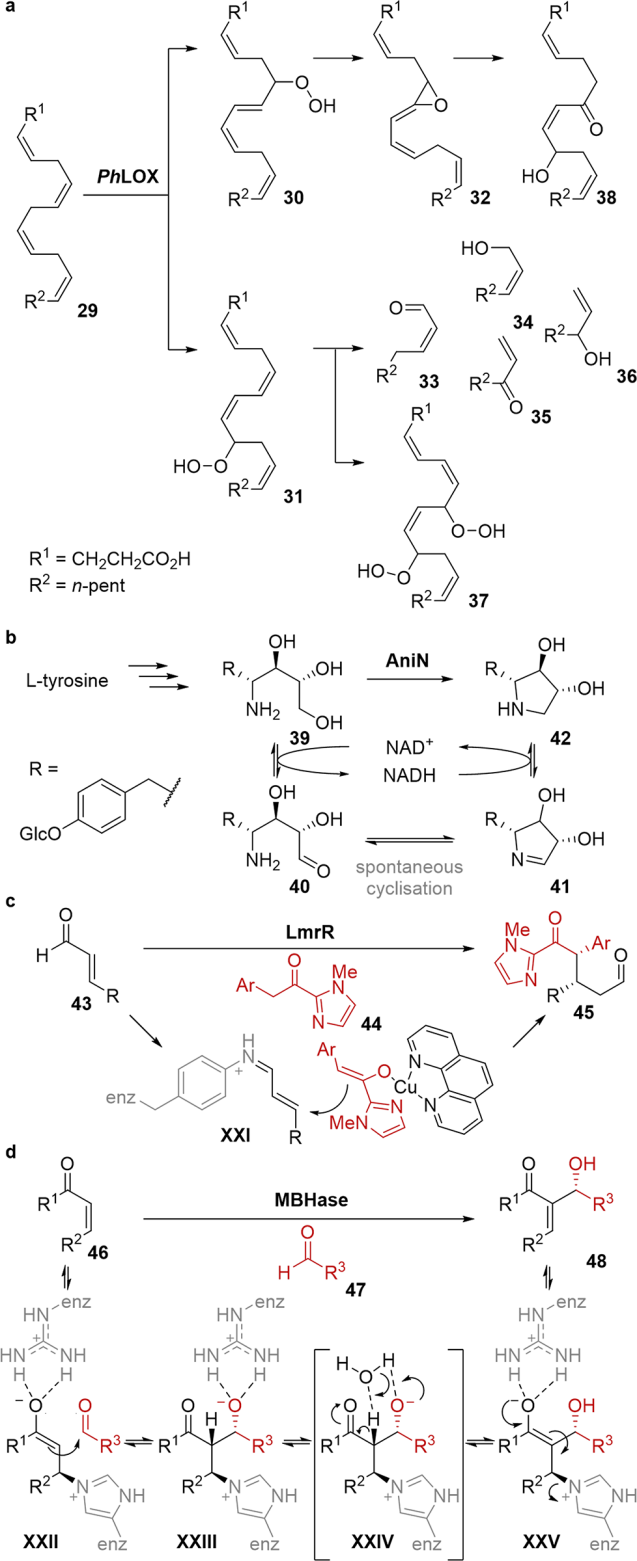
Examples of Multifunctional Enzymes in Metabolic Pathways and Designed Biocatalysts with Complex Mechanisms: (a) the Algal Lipoxygenase; (b) the Short-Chain Dehydrogenase AniN; (c) an Artificial Enzyme with Multiple Synergistic Abiological Active Sites; (d) an Enzyme for the Multistep Morita–Bayliss–Hillman Reaction

The presented examples clearly demonstrate that enzymes can possess the active site plasticity required to selectively bind as well as catalyze multiple different chemical reactions with structurally related substrates and often have broad substrate scope. These MFBs are derived from numerous sources, including engineered biocatalysts as well as wild-type sequences, and are found across multiple modes of substrate activation. The explanation for MFBs in nature is a subject of great speculation. A common understanding of evolutionary trajectory is that generalist enzymes are found deeper within a phylogenetic tree because lower order organisms, such as bacteria, tend to possess more primitive genomes that encode more promiscuous enzymes than higher order organisms.^43^ For example, a generalist and multifunctional enzyme that combines three activities was identified by Chen et al. while studying the evolution of a lower order algal lipoxygenase PhLOX (Scheme 6a).^44^ This evolutionary hypothesis is also supported by ancestral sequence reconstruction, which curates an artificial sequence space from multiple homologues alignments and claim that the number of specialist enzymes correlate with evolutionary time.^45−48^ This technique could offer a convenient method for creating new MFBs. Equally, MFBs that employ a single active site are common within biosynthesis including the well-known ATP citrate lyase reaction,^49^ as well as the remarkable 5-step macrofomate synthase reaction, and could be a source of inspiration.^50,51^ In anisomycin biosynthesis the multifunctional dehydrogenase AniN is capable of both alcohol oxidation and imine reduction (Scheme 6b),^52^ which in biocatalysis is well explored as a valuable hydrogen borrowing cascade using separate biocatalysts.^53,54^ More recently a single amine dehydrogenase, a so-called “alcohol aminase”, has been reported that catalyzes concurrent alcohol oxidation and reductive amination with ammonia.^55^ While this MFB only provided low yields, this enzyme could provide an excellent starting point for engineering an MFB for hydrogen borrowing.

As demonstrated by the identification of EneIREDs,^40^ metagenomics could offer opportunities to discover new MFBs by charting a vast and diverse sequence space.^41^ However, as enzyme function is often poorly annotated (not even considering for multifunctionality), identifying MFBs from large numbers of sequences could be challenging. To overcome this, rapid, selective and simultaneous screening technologies for multiple activities may need to be developed.^56^

Similar advances in screen development will be vital for MFB directed evolution campaigns to ensure multiple activities can be coevolved. In most of the featured examples, MFBs were engineered using a chromatographic screening strategy that can easily distinguish between single and multifunctionality but is generally low throughput. New approaches based on deep-learning and artificial intelligence^57^ will undoubtedly be advantageous for engineering MFBs because of the inherent mechanistic complexity created by multiple chemical reactions occurring within a single active site. Library design will also be critical as enhancing a subordinate activity or completely curating a further activity while maintaining the original reaction could present new and complex challenges. Advanced understanding of the reaction mechanism, access to good quality structural data for modeling, and reaction kinetics will be essential for successful MFB engineering.

Looking beyond engineering natural enzymes and toward *de novo* enzyme design, MFBs may lie on the horizon. Progressively more complex reaction mechanisms are being designed into protein scaffolds including bioconjugated multifunctional metal complexes, multiple and synergistic catalytic sites, and intricate modes for substrate activation.^58^ For example, an encoded unnatural aminophenylalanine residue and a supramolecularly bound Lewis acidic Cu(II) complex were introduced into the lactococcal multidrug resistance regulator, LmrR (Scheme 6c), for respective iminium activation of enal **43** and enolization of ketone **44** for biocatalytic Michael addition.^59^ This artificial enzyme could be considered an MFB as the protonation step also appears to be stereocontrolled by the enzyme. Furthermore, a computationally designed enzyme, BH32, was engineered using directed evolution for the multistep Morita–Bayliss–Hillman reaction^60^ (Scheme 6d). This enzyme employs a nucleophilic histidine for enone **46** activation and can catalyze C–C bond formation between aldehyde **47** and the intermediate enolate **XXII**. Further engineering or evaluation of this enzyme may reveal a variant that is an MFB that can also catalyze the proton transfer shown in species **XXIV** that is required for release of product **48**.

## Conclusion and Future Outlook

Recent reviews and perspectives in biocatalysis have highlighted the need for more streamlined reactions and smarter enzymes in the future.^61,62^ New MFBs would have a significant impact in these areas by lowering the number of biocatalysts and consequently reduce enzyme loading and engineering sequence space. These developments could mean a reduction in the cost of development and manufacture using enzymes and therefore accelerate the widespread adoption of biocatalysis in organic synthesis. The examples presented in this perspective demonstrate that MFBs are already simplifying biocatalytic routes across numerous enzyme families and substrate activation mechanisms to prepare a wide variety of complex stereoenriched functionalized products. New platforms for enzyme discovery and engineering (*e.g*., artificial intelligence, *in silico* designed enzymes, metagenomics) and new biocatalytic reaction modes (*e.g*., photobiocatalysis, carbene/nitrene chemistry, aminocatalysis) will undoubtedly expand the range of MFBs for organic synthesis and enable complex chemical transformations that will unlock routes to valuable molecules currently unknown in biocatalysis.
